# Editorial: Reviews in: ophthalmology 2022

**DOI:** 10.3389/fmed.2023.1255856

**Published:** 2023-07-31

**Authors:** Georgios D. Panos, Rashmi Deshmukh

**Affiliations:** ^1^Department of Ophthalmology, Queen's Medical Centre, Nottingham University Hospitals, Nottingham, United Kingdom; ^2^Division of Ophthalmology and Visual Sciences, Faculty of Medicine and Health Sciences, School of Medicine, University of Nottingham, Nottingham, United Kingdom; ^3^Cornea and Refractive Surgery Service, LV Prasad Eye Institute, Kallam Anji Reddy Campus, Hyderabad, India

**Keywords:** retina, cornea, ophthalmology, paediatric ophthalmology, COVID-19, ocular infections

## Introduction

Welcome to the Research Topic, “*Reviews in ophthalmology 2022*.” This issue is dedicated to providing a comprehensive overview of the latest advancements, challenges, and future directions in the field of ophthalmology. As we navigate through the third decade of the 21st century, it is clear that our understanding of ocular diseases and their management has significantly evolved, driven by groundbreaking research and technological innovations.

In this Research Topic, we have compiled a series of reviews that delve into various aspects of ophthalmology, from the surgical management of corneal disorders to the genetic underpinnings of complex ocular diseases. These reviews provide a snapshot of the current state of knowledge, highlighting the strides we have made and the challenges that lie ahead.

Each review in this Research Topic offers a unique perspective, reflecting the breadth and depth of research in ophthalmology. From exploring the effectiveness of different surgical techniques and treatment modalities to unravelling the genetic complexities of ocular diseases, these reviews represent the cutting edge of ophthalmic research.

As we present “*Reviews in ophthalmology 2022*,” we hope to foster a deeper understanding of the current landscape of ophthalmology, stimulate further research, and ultimately contribute to improving patient care. We invite you to delve into these insightful reviews, gain new knowledge, and join us in the ongoing quest to unravel the complexities of the human eye.

## Cornea and ocular surface

The field of ophthalmology has witnessed significant advancements in understanding and managing ocular surface diseases. This Research Topic summarises recent research on various ocular conditions, including ocular graft-vs.-host disease (oGVHD), Dry Eye Disease (DED), Keratoconus (KC), paediatric keratoconus, pterygium, and Fuchs' endothelial corneal dystrophy (FECD).

Salari et al. highlighted the effectiveness of superficial keratectomy (SK), a surgical procedure involving the manual dissection of the superficial layers of the cornea. The review emphasised the versatility of SK in addressing various ocular conditions, including corneal degenerations, dystrophies, scarring, recurrent corneal erosions, and retained corneal foreign bodies.

Tappeiner et al. focused on the challenges and concepts in diagnosing and managing oGVHD, a condition characterised by tissue inflammation following allogeneic hematopoietic cell transplantation. The review underscored the importance of interdisciplinary treatment approaches to improve patients' quality of life and prevent potentially irreversible visual loss.

Ling et al. discussed the increasing incidence of DED and the role of immune regulation defects in its pathogenesis. The review emphasised the need for anti-inflammatory drugs in treating moderate-to-severe DED and highlighted the potential of Traditional Chinese Medicine in managing the condition.

Hao et al. provided a comprehensive analysis of the pathogenesis of KC, an aetiologically heterogeneous corneal ectatic disorder. The study identified several genes and pathways involved in the disease's development, offering an integrated insight into the gene-based aetiology and pathogenesis of KC.

Li et al. conducted a meta-analysis comparing the efficacy of different corneal collagen cross linking (CXL) methods for paediatric keratoconus. The study concluded that standard epithelium-off CXL and accelerated epithelium-off CXL appear to be comparable in efficacy, with standard CXL providing greater changes in visual and pachymetric outcomes.

Taher, Alnabihi et al. performed a systematic review and meta-analysis on the management of primary pterygium, a common ocular surface disease. The study confirmed the effectiveness of a single intraoperative topical application of 0.02% mitomycin C during excision of pterygium followed by conjunctival autograft in reducing the rate of pterygium recurrence.

Lastly, Tsedilina et al. conducted a systematic review of the role of variants in the genes SLC4A11, ZEB1, LOXHD1, and AGBL1 in the development of FECD. The study confirmed the causal role of SLC4A11 variants in FECD, but further evidence is needed to confirm the roles of ZEB1, LOXHD1, and AGBL1 variants.

## Retina

In this Research Topic, we, also, explore the latest technological advancements, delve into the complex pathophysiological mechanisms, and discuss innovative therapeutic strategies that are shaping the future of retinal treatments.

Starting with Ladha et al.'s exploration of subretinal therapy, we are introduced to the potential of robotic technology in enhancing the precision and standardisation of ocular gene and cellular therapy delivery. The authors highlight the limitations of manual delivery, including the risk of iatrogenic damage and variability in delivery. They also underscore the importance of understanding the immune response elicited by the introduction of exogenous viral vectors or transplanted cells to the eye. The use of microprecision medical robotic technology is proposed as a solution to these challenges, offering reproducible and standardised delivery independent of injection speed.

Next, Tang et al. provide a comprehensive review of the development of risk factors and cytokines in Retinal Vein Occlusion (RVO), the second most prevalent retinal disease. The authors emphasise the complexity of RVO mechanisms due to the interrelated nature of risk factors. They also highlight the role of cytokines as powerful mediators of pathological conditions such as inflammation, neovascularisation, and macular oedema. This review underscores the need for continued research into the mechanisms and treatment targets of RVO.

Casciano et al. then delve into the role of the mammalian target of rapamycin (mTOR) pathway in diabetic retinopathy (DR). They outline how chronic hyperglycaemia can lead to retinal neurodegeneration through overactivation or inhibition of the mTOR pathway. The authors highlight the mTOR pathway's role in coordinating multiple anabolic and catabolic processes, such as autophagy, oxidative stress, cell death, and the release of pro-inflammatory cytokines. This review provides valuable insights into the potential of targeting the mTOR pathway in the management of DR.

Finally, Haydinger et al. provide a clinical overview of macular oedema, a complication of many retinal diseases that can lead to severe and permanent visual impairment and blindness. The authors discuss the mechanisms of disease, highlighting the dysregulation of the blood-retinal barrier as a key factor driving fluid accumulation in the central retina. They also discuss current treatments, including vascular endothelial growth factor blockers, corticosteroids, and non-steroidal anti-inflammatory drugs, and identify areas of opportunity for future research.

## Paediatric ophthalmology

In the realm of paediatric ophthalmology, two papers have made significant strides in understanding and treating conditions that affect the eyes of children.

The first paper, led by Gan et al., conducted a meta-analysis to evaluate the efficacy and safety of varying doses of atropine in slowing myopia progression in children. Myopia, or short-sightedness, is a common condition that affects a significant number of children worldwide. The study found that the efficacy and adverse effects of atropine are dose-dependent. High-dose atropine was found to be effective in slowing myopia progression, but its efficacy reduced after the first year of treatment. On the other hand, low-dose atropine showed better efficacy over a longer follow-up period. However, the higher the dose of atropine, the higher the incidence of adverse effects, such as photophobia. This meta-analysis provides valuable insights for clinicians in determining the appropriate dosage of atropine for treating myopia in children.

The second paper, by Taher, Ghaddaf et al., conducted a systematic review and meta-analysis to assess the efficacy and safety of intravitreal anti-vascular endothelial growth factor (anti-VEGF) injections for the treatment of retinopathy of prematurity (ROP). ROP is a potentially blinding eye disorder that primarily affects premature infants. The study found that anti-VEGF monotherapy was associated with fewer adverse events than laser therapy. However, there was no significant difference between the two treatments in terms of recurrence rate, treatment switching, retreatment, and mortality rate. This study provides a comprehensive review of the current standard treatment for ROP and offers valuable insights for future research and clinical practice.

## Ocular inflammations, infections, and COVID-19

The recent studies on the ocular implications of COVID-19 and other viral infections, as well as the effects of their respective vaccines, have shed light on a critical aspect of these pandemics that often goes unnoticed.

Zauli et al. highlighted the potential therapeutic role of the MDM2 inhibitor Nutlin-3 in protecting the eye from SARS-CoV-2 infection. The study suggests that the protein p53, present in high levels in the cornea, conjunctiva, and tear film, could play a protective role against the virus. The authors propose that the topical use of Nutlin-3 might protect the anterior surface of the eye from SARS-CoV-2 infection, thereby reducing the spread of the virus.

Akbari and Dourandeesh provided an updated overview of the ocular manifestations of COVID-19. The study emphasises the importance of paying attention to ocular manifestations during COVID-19, as they can be a presentation of life-threatening events such as stroke. Conjunctivitis is the most common presentation, which can develop at any stage of COVID-19, and there are also reports of life-threatening complications, such as rhino-orbital cerebral mucormycosis.

Taha et al. conducted a literature review highlighting the ocular complications of recent viral pandemics, including Monkeypox, SARS-CoV-2, MERS, Ebola, H1N1, and Zika viruses. The review also discusses the ocular complications of the vaccines and treatments used during these pandemics, and the role of the eye as a significant route of viral transmission.

Scalabrin et al. provided an overview of the ocular effects caused by viral infections and their corresponding vaccines, focusing on varicella zoster virus, measles virus, influenza viruses, hepatitis B virus, and SARS-CoV-2. The study aimed to establish a risk-benefit relationship from an ophthalmological point of view, comparing the pathological effects on the eye due to these viral infections with the possible ocular adverse effects of their respective vaccines.

Lastly, Abu-Ismail et al. discussed the impact of the COVID-19 pandemic and the wearing of face masks on ophthalmology practice. The study found that wearing face masks for long periods increases the chances of dry eyes and other ocular issues. The pandemic has also affected ophthalmology practices in managing patients, with new factors to consider, such as the risk of endophthalmitis, tests and symptoms of patients with glaucoma, and the emerging symptoms associated with the COVID-19 vaccination.

In conclusion, the reviews presented in this Research Topic provide a comprehensive overview of the current state of ophthalmology, highlighting the significant strides made in understanding and treating various ocular conditions. The reviews underscore the importance of continued research and innovation in addressing the challenges that lie ahead. The impact of the COVID-19 pandemic on ophthalmology, particularly the effects of prolonged face mask use, has emerged as a critical area of study. As we continue to navigate through these unprecedented times, it is crucial to adapt our practices and explore new avenues to ensure the best possible care for our patients. We hope that the insights provided in these reviews will stimulate further research and contribute to the advancement of ophthalmology.

## Author contributions

All authors listed have made a substantial, direct, and intellectual contribution to the work and approved it for publication.

